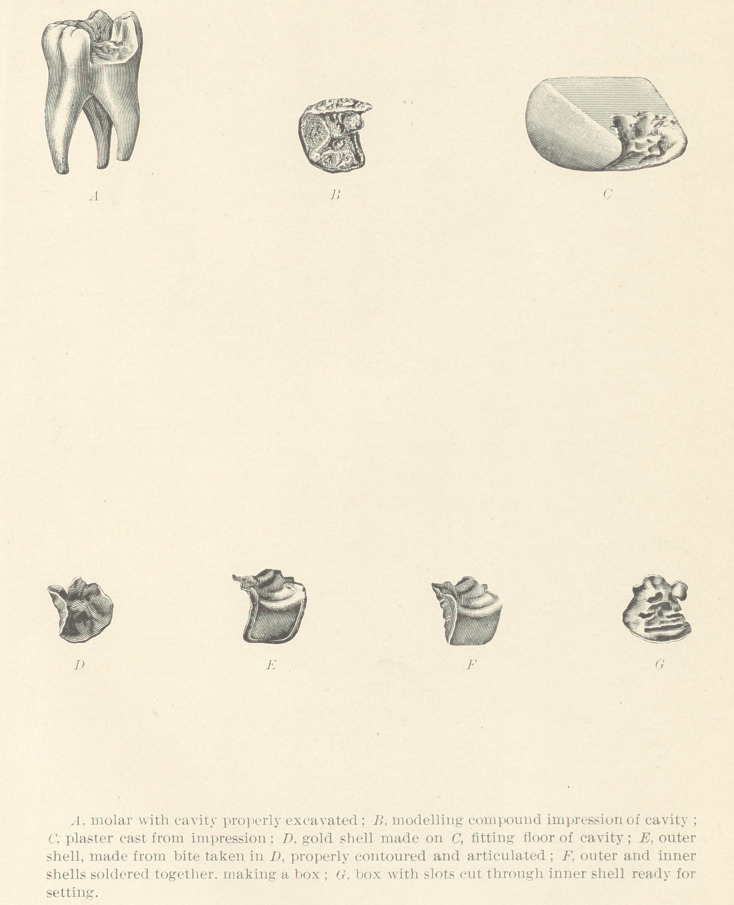# Boxings

**Published:** 1901-02

**Authors:** George S. Allan

**Affiliations:** New York


### International Dental Journal.

VOL. XXII. FEBRUARY, 1901. No. 2.

### Original Communications.[1]

### BOXINGS.[2]

BY DR. GEORGE S. ALLAN, NEW YORK.

WHAT I wish to bring to your attention will require but a short time to describe and fully illustrate. At a meeting held at Dr. Davenport's the first of last winter Drs. Dwight Smith and F. Milton Smith described their methods of using facings. The Drs. Smith showed at that meeting some models of the work they had done and at the same time gave an account of the service which the work had been to their patients and how gratifying the results had been to them.

This set me to thinking that possibly I had been remiss in my practice. I began to make facings with more or less success. The success was more than enough to compensate me for the trouble. After I had put in facings for a month or two, to my disgust I found now and then the edges were not perfect, and again, here and there, a facing came off. Still, there was a real merit in the method which I could not afford to drop. I kept try-

[1] The editor and publishers are not responsible for the views of authors of papers published in this department, nor for any claim to novelty, or otherwise, that may be made by them. No papers will be received for this department that have appeared in any other journal published in the country.

[2] Read before The New York Institute of Stomatology, November 9, 1900.

ing different methods, and finally took up porcelain. I had a great deal of satisfaction putting in inlays. It then came into my head, working at the two, that there might be a combination which would be satisfactory,—that is, something having the value and a great deal more strength than the porcelain.

My method consists of an inlay made of two shells of gold, an outer and an inner one, soldered together at the margins, the interior being filled with cement when in place. I make these inlays or boxes with the assistance of my laboratory man, and I think to do this work successfully and with little loss one should certainly have the help of an expert plate-worker. The first step is to prepare the cavity, filling the undercuts, if any, so that the impression-material will easily come out of it. It is necessary that the margins be especially prepared: they should be clean cut and smooth. I then take an impression of the cavity with modelling compound and hand the impression over to my plate-worker. I will here read a paper prepared by my plate-worker, giving his method of procedure after the impression leaves my hand.

"The model is made of plaster of Paris in the ordinary way. Use Mellotte's mouldine for making a mould of the cavity. Make a die of Mellotte's die-metal, using a piece of soft lead for the counter-die. Swage pure gold (30 American gauge) in the cavity. Great care must be taken in truing the gold to the exact margin of the cavity. This can be done in the mouth, or if the outlines are clear, it can be done on the die or model. Melt a small drop of solder in the bottom of the little cup or matrix, being careful not to let it flow on the margins. This will give it strength for handling. Burnish the cup in the cavity; see that the margins correspond with those of the cavity. Take an impression in wax or compound to get exact contour.

"The thickness of the gold must be allowed for in the wax, to avoid the bite striking too hard. A die is now made as before; 22-carat gold is used, as it will stand wear better than pure gold. The two pieces are soldered together, the edges trimmed and polished. A few saw-cuts in the pure gold inner shell will be sufficient for the cement to adhere."

After the inner shell has been returned from my mechanical man I place it in the cavity and burnish the edges carefully, seeing that they fit closely at every point. I then contour it out with

*A*, molar with cavity properly excavated; *B*, modelling compound impression of cavity; *C*, plaster cast from impression; *D*, gold shell made on *C*, fitting floor of cavity; *E*, outer shell, made from bite taken in *D*, properly contoured and articulated; *F*, outer and inner shells soldered together, making a box; *G*, box with slots cut through inner shell ready for setting.

wax, getting the proper shape and articulation, and return again to my plate-worker, who proceeds as he has described. When the finished box is returned to me, with a file I serrate the lower surface, at the time filing through this plate so that in setting the cement may be forced in, entirely filling the box. After the box has been filled, a small excess of cement is placed on this under surface, the boxing pressed home, and the excess of cement squeezed out. I believe this makes a filling more durable than a porcelain inlay because there are no thin edges that can be checked off, and there being no danger of breaking, however thin the box. It is better than a facing, because the margins are not liable to peel up, an objection I can urge to the facing. It is better than the all-gold inlay built up with solder, because with much less labor a perfect articulation is obtained without the grinding necessary in the case of an inlay of this kind or a porcelain inlay. I cannot state positively that this method is new; I only know that it is new so far as I am concerned.

## Figures and Tables

**Figure f1:**